# Periorbital Necrotizing Fasciitis: Presentation to Reconstruction

**DOI:** 10.7759/cureus.59501

**Published:** 2024-05-02

**Authors:** Kirupakaran Arun, Prachi Shah, Fiorella Grillon, Ian Subak-Sharpe

**Affiliations:** 1 Ophthalmology, Moorfields NHS Trust, London, GBR; 2 Ophthalmology, Whipps Cross Hospital, London, GBR

**Keywords:** necrotizing fasciitis, orbital cellulitis, group a streptococcus, debridement, periorbital, reconstruction

## Abstract

Periorbital necrotizing fasciitis (NF) is a devastating bacterial infection associated with irreversible inflammatory destruction of soft tissues. Outcomes include disfigurement, vision loss, septic shock, and death within hours to days. We describe two cases of periorbital NF that presented to our unit within a three-month period. We aim to highlight the key clinical features of periorbital NF, demonstrate the rapid progression of the disease, and the need for prompt identification and decisive intervention. Both patients presented with fever and left-sided periorbital swelling and showed rapid progression of swelling and gangrenous changes to the periorbital skin with worsening proptosis. They were treated with broad-spectrum intravenous antibiotics and underwent emergency surgical debridement of necrotic tissue followed by reconstruction. We propose a formal protocol that we recommend to aid the diagnosis and management of periorbital NF in an acute setting.

## Introduction

Necrotizing fasciitis (NF) is a rare, life-threatening infection that is characterized by a rapid progression from onset and significant destruction of skin and soft tissue [1]. NF most commonly affects the arms, legs, and perineum and these cases have a mortality rate of approximately 35% [2]. NF that affects the periorbital region is much rarer but reassuringly has a lower mortality rate of 3% [2]. This tends to be due to multiple factors such as an earlier presentation time to the hospital for treatment, the presence of the orbital septum that provides a barrier to posterior involvement, and the rich blood supply of the periocular skin that allows good antibiotic penetration.

Periorbital NF commonly presents with fever, periocular skin erythema or darkening proptosis, and significant pain [3]. Its clinical diagnosis and initial management should not be delayed for imaging purposes. Imaging, when performed, often demonstrates thickening of the fascia layers due to soft tissue edema and whilst the presence of gas is characteristic, it may be lacking [4]. MRI is the most sensitive investigation for identifying gas and CT is useful in determining the extent of the involvement of the disease [5].

As soon as periorbital NF is suspected, the initial treatment should be in the form of intravenous (IV) broad-spectrum antibiotics such as a combination of tazocin and clindamycin. If the disease is restricted to the eyelids, antibiotics alone and close monitoring may be sufficient [6]. However, if there is any suggestion of post-septal involvement or worsening of the clinical examination, urgent surgical debridement of necrotic tissue should be performed.

We present two cases of periorbital NF that highlight the rapid progression of this condition and emphasize the importance of early diagnosis. Once a diagnosis of periorbital NF is suspected, immediate surgical debridement should be performed. A multi-disciplinary approach is required as the extent of the spread of the infection can be difficult to ascertain pre-operatively. We describe the debridement and washout procedure that we employed and emphasize the importance of close post-operative monitoring to plan appropriate reconstruction.

## Case presentation

Case 1

**Figure 1:**
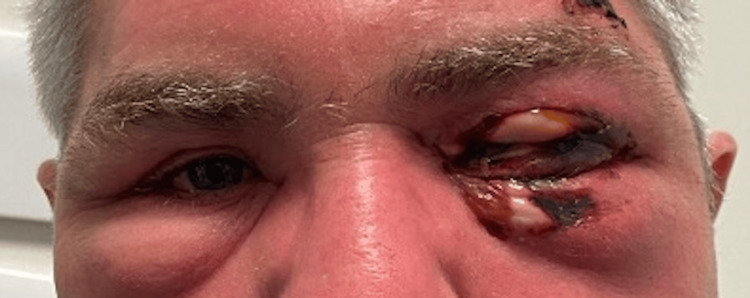
Bilateral periorbital swelling with left patches of skin necrosis. Area of initial laceration seen superior to left eyebrow

A 52-year-old male with no past medical history sustained blunt trauma with a wooden beam to his left forehead (just above his eyebrow) following an accidental fall whilst at home. He reported no loss of consciousness, no headache, and only superficial bleeding from the laceration site that resolved with pressure within five minutes. Forty-eight hours later, the patient developed rapid progressive left periorbital swelling and proptosis and experienced five episodes of vomiting before self-presenting to the emergency department. By this point, there were signs of tense left periorbital swelling with the patient unable to open his eyelids. The swelling had started to track over the bridge of the nose to involve the right periorbital area. There were also signs of irregular white and black skin pigmentation of the left upper and lower lids suggestive of skin necrosis (Figure 1).

Initial observations included a temperature of 38.7^o^C, a heart rate of 72, and a blood pressure of 134/82 mmHg. Blood tests showed raised inflammatory markers with a white cell count (WCC) of 11 x 10^9^/L, C-reactive protein (CRP) of 71 mg/L, and a blood lactate of 5.8 mmol/L. He was immediately given a dose of IV co-amoxiclav. CT head and orbits showed extensive left periorbital swelling with subcutaneous fat stranding and fluid collections within the left periorbital pretarsal spaces and a working diagnosis of peri-orbital cellulitis was made.

Three hours later, repeat blood tests were taken which showed a dramatic worsening of his infection markers. The WCC had risen to 17.3 x 10^9^/L and the CRP had jumped to 515 mg/L. The patient also complained of worsening left periorbital pain. On clinical examination, the left periorbital region felt more tense with an inability to open the eyelids even manually. Due to the rapid deterioration, we began to suspect periorbital NF as the top differential. A decision was made for urgent debridement and antibiotics were changed to IV tazocin 3x/day and IV clindamycin 4x/day with a STAT dose of IV amikacin.

**Figure 2:**
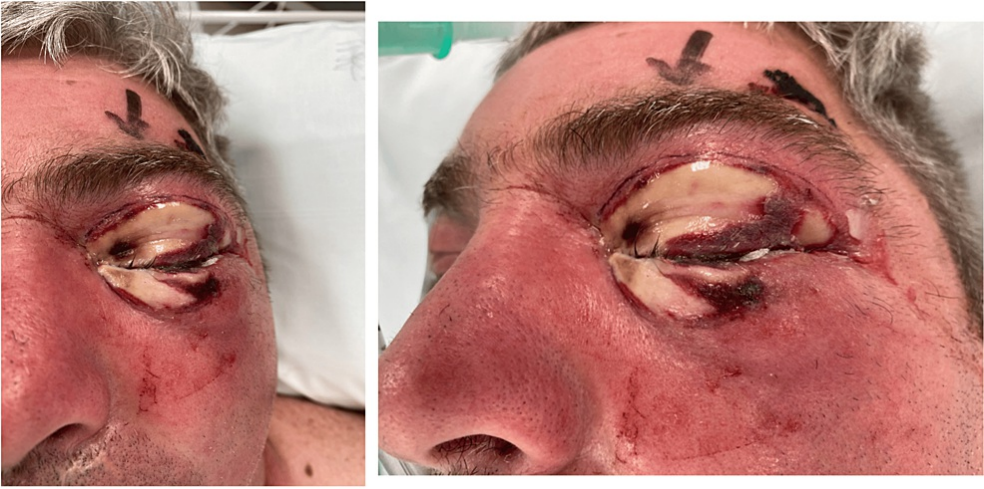
Significant extension of left periorbital necrosis. Photo taken six hours after Figure 1

Prior to debridement, due to the suspected orbital compartment syndrome from the tense left periorbital swelling, a lateral canthotomy was performed under local anesthetic. On the immediate release of the lateral canthal tendon, there was significant yellow pus seen discharging from the lateral orbit. Following lateral canthotomy, the patient consented to urgent debridement and it was noted at this time that the left periorbital skin necrosis had significantly extended from the initial presentation six hours earlier to involve the entire upper lid and over 50% of the lower lid (Figure 2).

Debridement was performed under a general anesthetic. Debridement of the upper and lower lid necrotic skin was performed by the ophthalmology team. Significant pus and white necrotic matter were found in the upper and lower eyelids and sent for histopathology, microbial culture, and sensitivities. The underlying fascial planes, orbicularis oculi, orbital septum, and part of the levator complex appeared white with minimal bleeding (suggesting tissue necrosis) and were debrided. Further exploration along the fascial planes of the cheek was carried out in conjunction with the maxillo-facial team until the tissue appeared viable. The debrided areas were washed several times with large quantities of normal saline and hydrogen peroxide.

**Figure 3:**
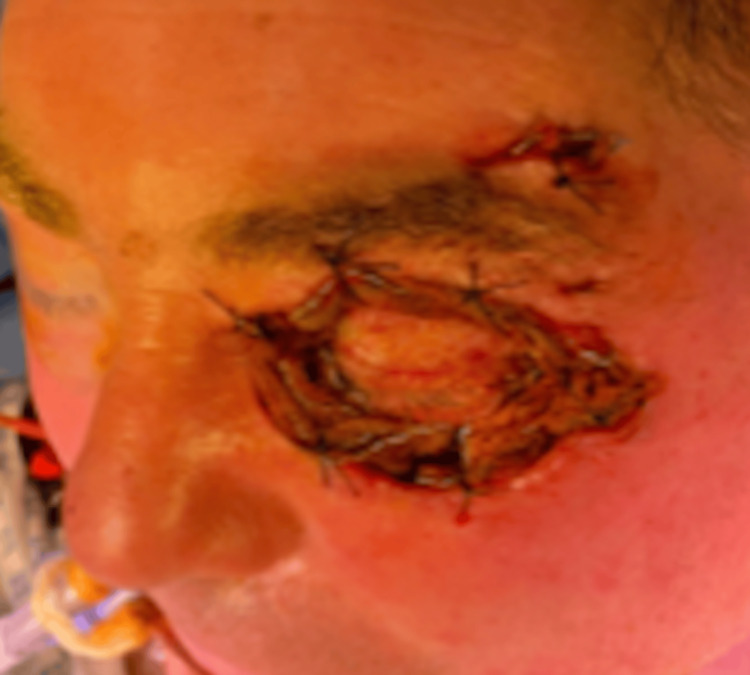
Clinical photograph taken immediately following debridement. Iodine-soaked gauze seen packed in the debrided left periorbital region

Following adequate exploration and debridement of all necrotic appearing tissue, the debrided area was packed with iodine-soaked gauze which was sutured in place (Figure 3). The extent of skin erythema following surgery was marked with a pen. The patient was intubated and transferred to the intensive care unit (ICU) for post-operative monitoring.

The patient was reviewed two hours following surgery and thereafter daily in the ICU. After 48 hours there was a clear improvement in vital signs and bloods. He did not spike any temperatures and his WCC came down to 11.8 x 10^9^/L (compared to 17.3 x 10^9^/L prior to debridement) and the CRP came down to 23 mg/L (compared to 515 mg/L prior to debridement). Cultures sent from the pus and necrotic tissue grew group A Streptococcus.

**Figure 4:**
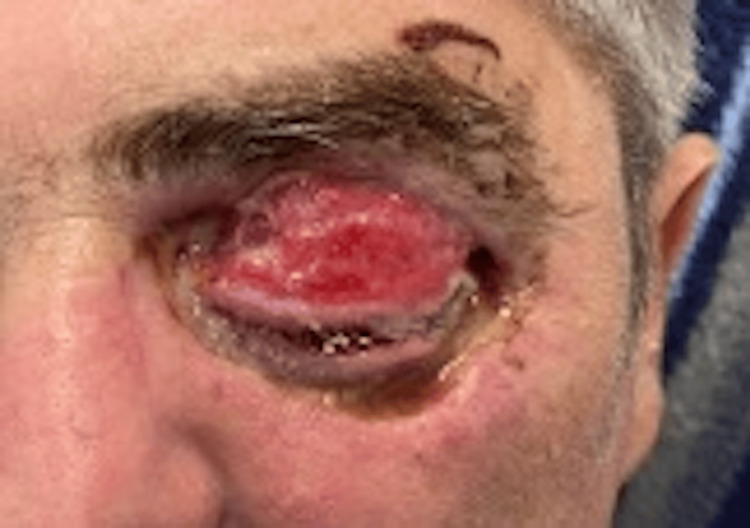
Well-healed lower lid with pink granulation tissue formed over the upper lid. Photo taken one-week following debridement

After 72 hours, the iodine-soaked gauze was removed and the patient was extubated. He completed a seven-day course of IV tazoin and clindamycin. At this point, there were signs of granulation tissue forming at the healing wounds (Figure 4). His vision from the left eye was 6/36 unaided with no signs of a relative afferent pupillary defect. Eye movements were full in all directions except abduction. There was a reduced lid opening with a 4 mm levator function.

The patient was scheduled for upper and lower lid reconstruction two weeks after debridement. This was canceled on the day of surgery as the patient tested positive for COVID-19 and was instead performed one week later. Skin grafts were taken from both the supraclavicular regions and the left post-auricular region.

**Figure 5:**
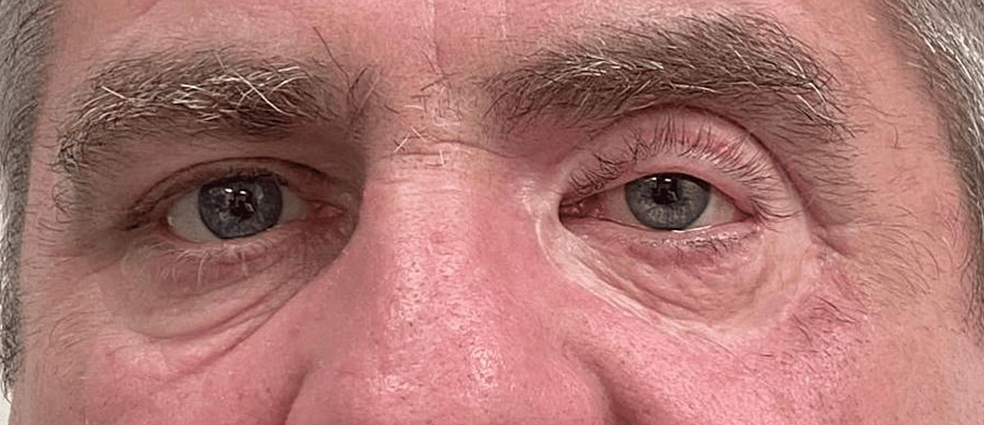
Photograph demonstrating good voluntary lid opening following reconstruction with skin grafts

Three months following surgery, the patient was able to achieve 20/40 unaided visual acuity in the left eye. There was complete voluntary closure of his eyelids with a maximal 7 mm lid opening and the patient was happy with the cosmetic and functional outcome (Figure 5). Since then he has had no further periocular infections.

Case 2

An 80-year-old gentleman presented to the emergency department with a fever (38.6^o^C) and a 24-hour history of left periorbital swelling. His past medical history included rheumatoid arthritis, for which he was having rituximab infusions.

**Figure 6:**
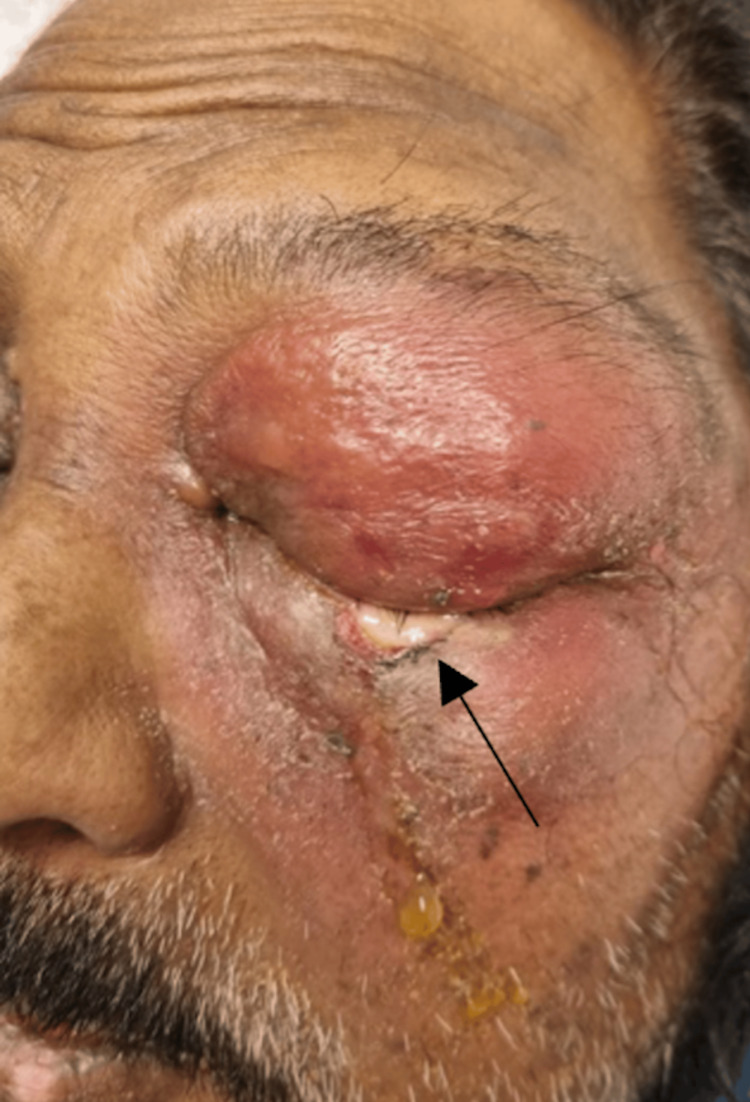
Left periorbital swelling with early skin necrosis (black arrow)

On initial presentation, there was a patch of whitening of the left lower lid skin that was discharging yellow pus (Figure 6). A sample of the pus was sent for microscopy, culture, and sensitivity (MC&S).

Ocular examination demonstrated a 2 mm voluntary lid opening. On lifting the eyelid manually, his left pupil was equal in size to the right pupil with equal responses to light and accommodation on both sides. There was no relative afferent pupillary defect. His eye movements demonstrated full elevation, depression, and adduction with a moderate reduction in abduction.

Blood tests showed raised inflammatory markers with a WCC of 20.9 x 10^9^/L and a CRP of 305 mg/L. He was immediately given a dose of IV co-amoxiclav.

At this point, a diagnosis of periorbital NF was immediately suspected and the patient was listed for emergency debridement of necrotic tissue. He was started on IV tazocin and IV clindamycin following a discussion with microbiology.

The debridement was performed under a general anesthetic. Necrotic skin and underlying fascia were debrided and excised until healthy tissue was noted at all margins. Once all the necrotic tissue had been excised, pockets were created along the margins in a deep plane and washed with large quantities of normal saline. The wounds were covered with chloramphenicol ointment, jelonet, and iodine-soaked gauze.

**Figure 7:**
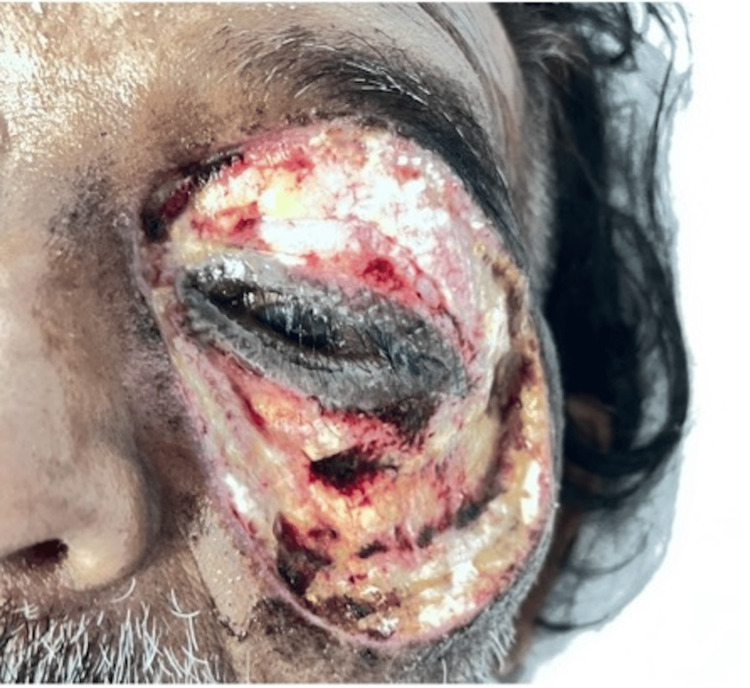
Day 1 post debridement demonstrating early granulation tissue following removal of iodine-soaked gauze

The patient was reviewed eight hours following debridement. The wound margins showed no signs of residual disease with early signs of healthy granulation tissue (Figure 7).

The pus MC&S came back with the growth of group A Streptococcus (sensitive to penicillin and clindamycin). IV antibiotics were accordingly changed to benzylpenicillin and clindamycin. The wound margins healed nicely and infection markers returned to normal values within 96 hours of debridement.

**Figure 8:**
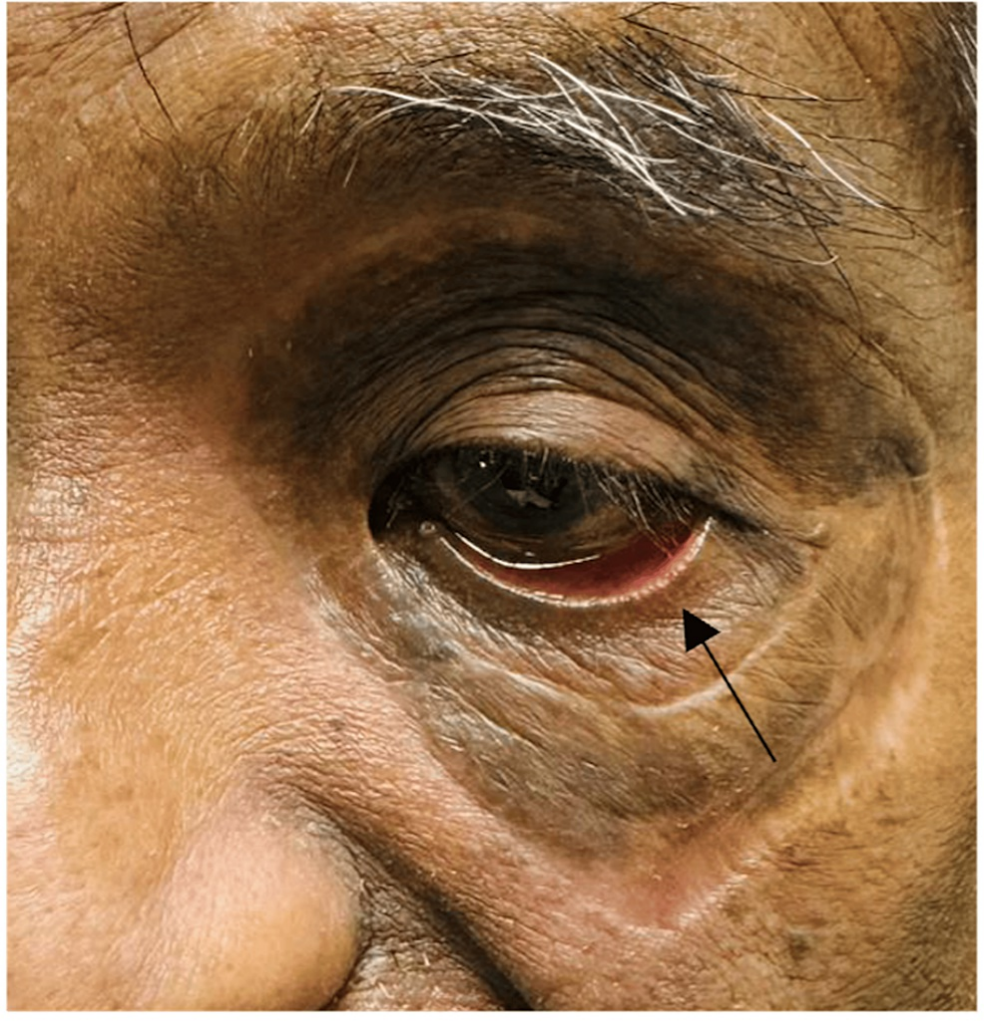
Left lower lid cicatricial ectropion (black arrow) following contraction of scar tissue

Reconstruction of the left periorbital region was performed two weeks following debridement. Skin grafts were harvested from the left supraclavicular region (for the left upper lid) and right upper inner arm (for the left lower lid). The skin grafts healed very nicely over the next few months with no signs of graft necrosis but he started to develop a left lower lid cicatricial ectropion secondary to contraction of scar tissue (Figure 8).

He underwent successful surgery to correct this ectropion with further skin grafting of the left lower lid. The skin was harvested from the right supraclavicular region for this. The lower lid remained in a good position with full eyelid closure and best-corrected vision in this eye was recorded as 20/30.

## Discussion

NF is a potentially fatal infection of the skin, superficial fascia, and subcutaneous tissue. It can be characterized into three stages, although in clinical practice the rapidly progressive nature of the condition may mean the stages can blur into one [7]. Stage 1 involves localized edema, fever, and pain out of proportion to the other signs. Stage 2 involves localized tissue ischemia that is thought to occur due to thrombosis of the vasculature and clinically the skin starts to show small blisters. Stage 3 represents a complete blockage of the blood supply to the fascia and results in loss of sensation to that area, patchy discoloration of the overlying skin, and gas production seen on imaging.

Both of our patients progressed from stage 1 on presentation to stage 3 within 6-12 hours. We stress this to emphasize how early diagnosis is absolutely critical to minimizing morbidity and mortality. Risk factors to look out for in the history that predisposes to NF include immunosuppression, malnutrition, prior trauma or surgery to the affected region, liver disease, and diabetes [8,9].

NF can be classified based on the responsible organism [10]. Type 1 NF occurs in immunocompromised patients and often is caused by a combination of aerobic and anaerobic organisms. Type 2 NF is caused by Staphylococcus or Streptococcus organisms, with group A Streptococcus being the most common. Both of our patients had Type 2 NF, confirmed from the culture results and this is much more common for periorbital NF.

A multidisciplinary approach is essential to providing optimal patient care for a patient with NF [11]. In our cases, multidisciplinary input was sought from ophthalmology, microbiology, general surgery, maxillo-facial, intensive care, and psychiatry. In our first case, prior to debridement, a lateral canthotomy was performed due to the tense orbit on examination giving concern to possible orbital compartment syndrome. This can also be performed intra-operatively during debridement to help with access. However, if there are concerns regarding raised intra-orbital pressure it is good practice to not delay performing a lateral canthotomy. The main signs to suggest this would be raised intraocular pressure, proptosis, and any signs of optic nerve compromise such as pain in eye movements, a positive relative afferent pupillary defect, and/or sudden vision loss [12]. In our patient, the periorbital swelling did not allow any eyelid opening and as such the only quantifiable sign we could rely on was the proptosis and manual palpation of the globe through the lids to estimate intraocular pressure.

Almost all cases of periorbital NF undergo surgical debridement due to the often delayed diagnosis [13]. Any suspected necrotic tissue can be easily identified by the discoloration of the tissue (often white) with minimal bleeding due to the ischemia. The boundaries of the debridement should ideally include healthy, bleeding tissue [14]. During debridement, tissue should be sent for histopathology and broad-spectrum IV antibiotics should be continued [15].

**Figure 9:**
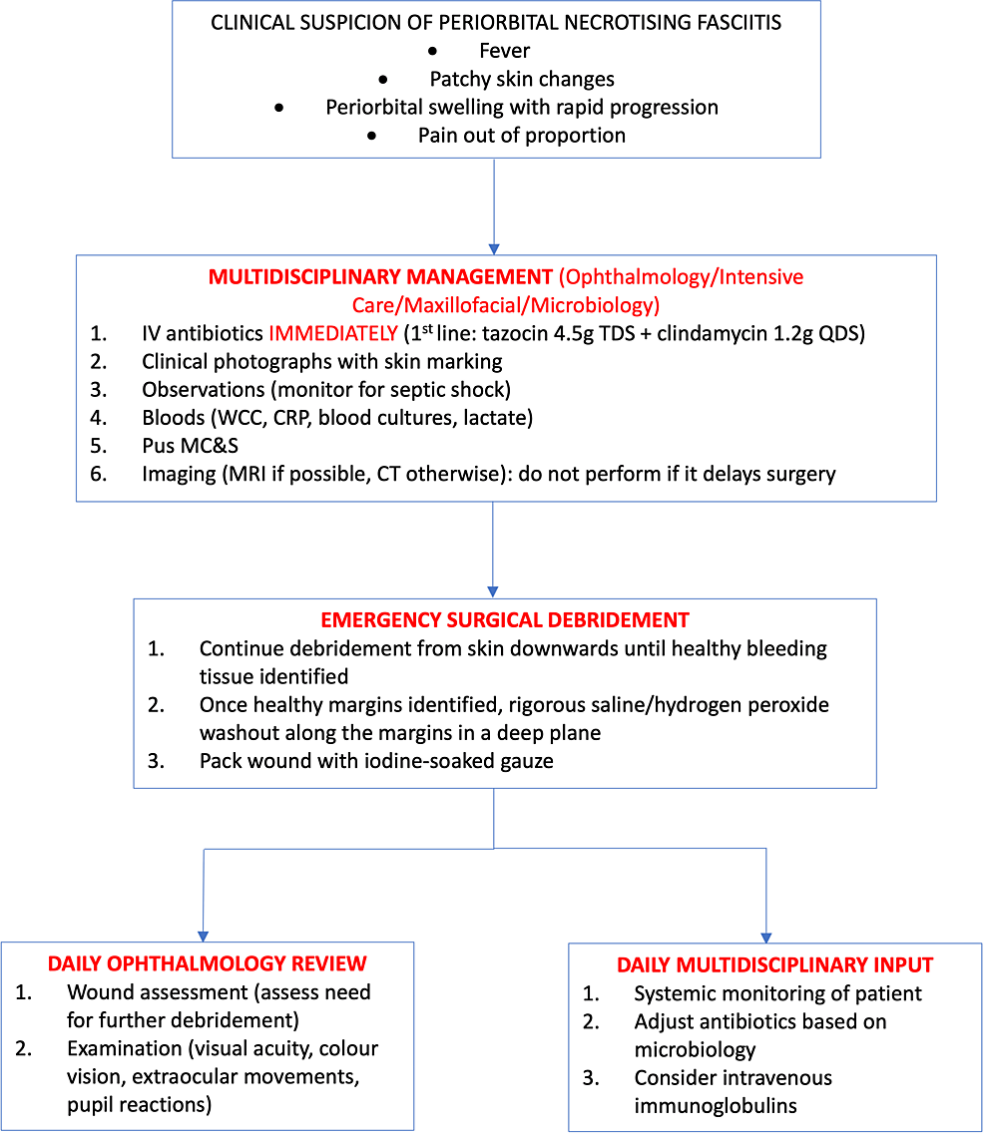
Algorithm for the acute management of a patient with suspected periorbital necrotizing fasciitis IV: intravenous; TDS: three times a day; QDS: four times a day; WCC: white cell count; CRP: C-reactive protein; MC&S: microbial culture and sensitivities; MRI: magnetic resonance imaging; CT: computed tomography

Based on our experience, we propose a protocol to consider for the acute management of periorbital NF (Figure 9).

Following the resolution of the active infection, reconstruction should ideally be performed as soon as possible. The aim of this is to provide a good cosmetic and functional outcome but it should be stressed to the patient that multiple procedures may be required and throughout the clinical course psychosocial support to the patient is essential.

## Conclusions

Periorbital NF is one of the potentially life-threatening diseases that ophthalmologists can come across. It is very rare and orbital cellulitis has similar presenting symptoms; hence, the diagnosis of periorbital NF is often delayed. It is essential for ophthalmologists and emergency doctors to be able to recognize and manage periorbital NF. Whilst the diagnosis is mainly clinical, imaging and blood markers support the diagnosis and aid in monitoring response to treatment. A multidisciplinary approach to the management of these patients is essential and the seriousness of the condition should be stressed to the patient and family as well as the need for long-term follow-up and the potential complications associated with NF.
